# Safety and efficacy of high-dose daptomycin as salvage therapy for severe gram-positive bacterial sepsis in hospitalized adult patients

**DOI:** 10.1186/1471-2334-13-66

**Published:** 2013-02-04

**Authors:** Chung-Chih Lai, Wang-Huei Sheng, Jann-Tay Wang, Aristine Cheng, Yu-Chung Chuang, Yee-Chun Chen, Shan-Chwen Chang

**Affiliations:** 1 Department of Internal Medicine, Kaohsiung Medical University Hospital, Kaohsiung, Taiwan; 2 Department of Internal Medicine, National Taiwan University Hospital, Taipei, Taiwan; 3 Department of Internal Medicine, Far-East Memorial Hospital, New Taipei City, Taiwan; 4 Division of Infectious Diseases, Department of Internal Medicine, National Taiwan University Hospital, 7 Chung-Shan South Road, Taipei, 100, Taiwan

**Keywords:** Daptomycin, High dose, Creatine phosphokinase, Treatment outcomes

## Abstract

**Background:**

Increasing the dosage of daptomycin may be advantageous in severe infection by enhancing bactericidal activity and pharmacodynamics. However, clinical data on using daptomycin at doses above 6 mg/kg in Asian population are limited.

**Methods:**

A retrospective observational cohort study of all hospitalized adult patients treated with daptomycin (> 6 mg/kg) for at least 72 hours was performed in Taiwan.

**Results:**

A total of 67 patients (40 males) with a median age of 57 years received a median dose of 7.61 mg/kg (range, 6.03-11.53 mg/kg) of daptomycin for a median duration of 14 days (range, 3–53 days). Forty-one patients (61.2%) were in intensive care units (ICU). Sites of infections included complicated skin and soft tissue infections (n = 16), catheter-related bacteremia (n = 16), endocarditis (n = 11), primary bacteremia (n = 10), osteomyelitis and septic arthritis (n = 9), and miscellaneous (n = 5). The median Pitt bacteremia score among the 54 (80.6%) patients with bacteremia was 4. The most common pathogen was methicillin-resistant *Staphylococcus aureus* (n = 38). Fifty-nine patients (88.1%) were treated with daptomycin after glycopepetide use. Overall, 52 (77.6%) patients achieved clinical success. The all-cause mortality rate at 28 day was 35.8%. In multivariate analysis, the significant predictors of in-hospital mortality in 54 bacteremic patients were malignancies (*P* = 0.01) and ICU stay (*P* = 0.02). Adverse effects of daptomycin were generally well-tolerated, leading to discontinuation in 3 patients. Daptomycin-related creatine phosphokinase (CPK) elevations were observed in 4 patients, and all received doses > 8 mg/kg.

**Conclusions:**

Treatment with high dose daptomycin as salvage therapy was generally effective and safe in Taiwan. CPK level elevations were more frequent in patients with dose > 8 mg/kg.

## Background

Daptomycin, a cyclic lipopeptide antibiotic, has a rapid, concentration-dependent bactericidal activity against clinically relevant gram-positive organisms, such as *Staphylococcus aureus* (including methicillin-resistant *S. aureus*, MRSA) and enterococci (including vancomycin-resistant enterococci, VRE). The approved dose of daptomycin is 4 mg/kg for the treatment of complicated skin and skin structure infections and 6 mg/kg for *S. aureus* bacteremia, including right-sided infective endocarditis [1,2]. Creatine phosphokinase (CPK) level elevation with associated muscular symptoms, has been a concern during daptomycin treatment [3].

Among patients suffering from infections in the intensive care units (ICU), *S. aureus,* predominantly MRSA, remains one of the most common causative organism (up to 20%) [4]. *S. aureus* isolates with reduced daptomycin susceptibility, especially after receiving prior glycopeptitide therapy, have been described and were associated with clinical failure [5-7]. Increased activity with higher doses, especially against isolates with reduced susceptibility, was demonstrated in some *in vitro* and animal studies [8-10]. The area under the plasma concentration time curve (AUC) and trough level of daptomycin increased proportionally with escalating doses [11]. Thus, increasing doses of daptomycin seems attractive for severe infections, critically ill patients, or prior treatment failure with glycopeptides [12]. The experience with high dose daptomycin (> 6 mg/kg) is still limited worldwide, and it is unclear whether this strategy improves outcomes [13-17].

Previous data in Taiwan demonstrated similar pharmacokinetics profiles of daptomycin between Taiwanese and Caucasian population, and supported daptomycin as a promising and safe agent for severe staphylococcal infections [18,19]. Our objective in this study was to evaluate the clinical efficacy and safety of high-dose daptomycin for serious infections among adult patients, especially among those pretreated with other anti-gram positive antimicrobial agents.

## Methods

### Patient enrollment

This retrospective observational cohort included hospitalized patients who were treated with intravenous daptomycin (Cubicin, Cubist Pharmaceuticals, Lexington, MA) at the National Taiwan University Hospital (NTUH), a tertiary medical center of 2200 beds, from September 2009 to October 2010. Audit by an infectious diseases physician is necessary to prescribe daptomycin at NTUH, therefore all patients receiving daptomycin during the study period could be identified at onset of therapy. To be eligible, patients had to be more than 18 years old and received daptomycin intravenously at doses more than 6 mg per kilogram of body weight for at least 72 hours. The dosage and duration of daptomycin were determined by the physicians in charge and consulting infectious diseases specialists (Lai CC, Sheng WH, and Wang JT). Daptomycin dosing was based on mg per kg of actual body weight. The intervals were every 24 hours for patients with creatinine clearance >30 mL/min and every 48 hours for patients with creatinine clearance below 30 mL/min, respectively. The maximal dosing of daptomycin was 12 mg/kg/day [11].

The definitions of high-dose daptomycin were diverse. Both 6 and 8 mg/kg have been used as the cut-off values in the literature [13,14,16,17]. Based on the preparation of daptomycin is 500 mg per vial in Taiwan and an average body weight of 60 kilogram among Taiwanese adults, we divided our study cohort (all treated with doses more than 6 mg/kg) into two groups based on daptomycin doses (> 8 mg/kg or ≤ 8 mg/kg) and evaluated their safety and efficacy separately.

### Ethics statement

This research conformed to the Helsinki Declaration and local legislation, and was approved by the National Taiwan University Hospital Research Ethics Committee (No. NTUH-200908009R). The committee waived the need for written informed consent for participation in the study because of the observational nature of the study design and retrospective data collection. The clinical samples in the study were not collected for research purposes, but as part of standard care.

### Data collection

A standard case record form was used for data collection. Clinical data were collected from their medical records, including age, sex, underlying diseases, clinical diagnosis, microbiologic isolate identification and antibiotic susceptibility, indication for daptomycin use, use of sequential or concurrent antibiotics, dose and duration of daptomycin, and adverse events. CPK levels were checked at least once per week during treatment with daptomycin based on infectious diseases physicians’ recommendation. Creatinine clearance was calculated at baseline according to the Cockcroft–Gault formula. The Charlson comorbidity index was used to evaluate comorbidity conditions of the patients [20]. The clinical severities were assessed by the Pitt bacteremia scores [21]. Sequential Organ Failure Assessment (SOFA) scores were calculated for ICU patients within 24 hours of initiation of high-dose daptomycin therapy.

### Bacterial culture and drug susceptibility tests

Culture, identification, and susceptibility testing of clinical isolates were performed according to standard microbiological methods [22]. BACTEC (Becton Dickinson, Spark, MD, USA) automated bacterial blood culture systems were used to facilitate rapid identification. Other clinical specimens were plated onto a sheep blood agar (SBA) plate. *S. aureus* isolates were spotted onto ChromAgar MRSA to check for methicillin resistance [23]. The susceptibilities of MRSA isolates to vancomycin were determined as minimum inhibitory concentrations (MICs) using Etest (AB Biodisk, Solna, Sweden) [24].

### Outcomes and safety evaluation

All patients were followed until death or discharge. Outcomes were evaluated at the time of 14 days and 30 days after daptomycin start, and at the end of hospitalization. Clinical success was defined as resolution of the signs and symptoms of attributed infections during treatment with daptomycin. Microbiological success was defined as the eradication or presumed eradication of infecting pathogens at sites of infection [13,16]. The following conditions was also counted as clinical failure: (1) infection-related death, (2) persistence of the signs or symptoms of infection after initiating 72 hours of therapy with daptomycin, (3) relapse or recurrence of infections within 30 days after discharges, or (4) replacement of daptomycin with other anti-gram positive antimicrobial agents. Persistent positive cultures from blood or infection sites after 72 hours after receiving daptomycin were counted as microbiological failure [13]. Infection-related death was defined as (1) persistent isolation of pathogens from the infection sites at death, (2) death before resolution of symptoms and signs of infection, or (3) death within 14 days after the onset of infection without other attributable causes [25].

Elevations of CPK level were defined as (1) CPK values ≥ 3 times the upper limit of normal (ULN) based on two serial measurements during therapy, and one of two levels ≥ 5 times the ULN or (2) CPK levels ≥ 5 times the ULN on two serial measurements if abnormal CPK levels at baseline [26]. The ULN of CPK value at NTUH is 160 IU/L.

### Statistical analyses

SPSS software was used to analyze the data (IBM Corporation. NY, USA). Categorical data were analyzed using chi-square or Fisher’s exact tests, as appropriate, and continuous variables were compared using the Wilcoxon test. A *P* value of < 0.05 was considered significant (two-tailed test). A multivariate model was used to analyze the predictors of in-hospital mortality among bacteremic patients receiving daptomycin therapy.

## Results

During the study period, a total of 67 hospitalized adult patients had documented gram-positive bacterial infections and received high-dose daptomycin therapy for more than 72 hours. The demographic data are shown in Table 1. Nearly 70% of episodes were attributable to healthcare-associated infections. Over one fourth of patients had renal failure and required renal replacement therapy during hospitalization. Hematologic and solid organ malignancies were present in one third (34.3%) of patients. During treatment with daptomycin, 41 (61.2%) patients had been hospitalized in ICU, with a median SOFA score of 7 (interquartile range, 4–12). Thirty-three of 67 patients (49.3%) required ventilator support when receiving daptomycin therapy.

**Table 1:** Clinical characteristics of 67 patients with high-dose daptomycin therapy

**Characteristic**^**a**^	**Daptomycin dose (mg/kg)**		
	≤** 8 (n = 41)**	>**8 (n = 26)**	***P *value**
Age, years	56 (18–89)	58.5 (22–87)	0.57
Male (%)	23 (56.1%)	17 (65.4%)	0.45
Weight, kg	56 (37–89)	61.5 (42–87)	0.13
Creatinine clearance, mL/min	38 (5–263)	57.5 (10–210)	0.54
Indwelling catheter use^b^	31 (75.6%)	16 (61.5%)	0.22
Surgery within 3 months	13 (31.7%)	10 (38.5%)	0.57
Underlying diseases			
Diabetes mellitus	14 (34.1%)	8 (30.8%)	0.77
Cirrhosis of liver	1 (2.4%)	4 (15.4%)	0.14
ESRD	11 (26.8%)	7 (26.9%)	0.99
Heart failure	11 (26.8%)	11 (42.3%)	0.19
Chronic lung disease	5 (12.2%)	3 (11.5%)	0.94
Malignancy	17 (41.5%)	6 (23.1%)	0.12
Charlson index	4 (0–11)	3.5 (0–11)	0.46
Pitt bacteremia score^c^	4 (0–10)	3 (0–7)	0.85
ICU stay	24 (58.5%)	17 (65.4%)	0.58
SOFA score^d^	7 (2–17)	8 (0–17)	0.49
Ventilator use	19 (46.3%)	14 (53.8%)	0.55
Previous anti-gram positive antibiotic			
Vancomycin	28 (68.3%)	22 (84.6%)	0.14
Teicoplanin	13 (31.7%)	9 (34.6%)	0.81
Linezolid	5 (12.2%)	4 (15.4%)	0.71

^a^ Data are median (range) for continuous variables.

^b^ Including central venous catheter, port-A catheter, Hickman catheter, temporal pacemaker, and intra-aortic balloon pump.

^c^ Only for bacteremic patients.

^d^ Only for ICU patients.

*ESRD*, end-stage renal disease; *ICU*, intensive care unit; *SOFA*, Sequential Organ Failure Assessment; *VHD*, valvular heart disease.

The most common causative pathogen was MRSA (56.7%), followed by VRE (26.9%) and methicillin-resistant coagulase-negative staphylococci (MRCoNS) (14.9%) (Table 2). MIC of vancomycin was checked in 31 of stored MRSA isolates, and MIC ≥ 2 μg/mL were found in 24 (77.4%) of them. The median of MIC of vancomycin of MRSA isolates was 2 μg/mL with a range from 1 μg/mL to 3 μg/mL. Complicated skin and soft tissue infections (23.9%) and indwelling catheter-related bacteremia (23.9%) were the most common infections. Among 11 patients with endocarditis, 10 were left-sided and one was right-sided. Fifty-four patients (80.6%) were bacteremic, and the median Pitt bacteremia score was 4 (range, 0–10) (Table 1).

**Table 2:** Types of infections, pathogens and dosage of 67 patients with high-dose daptomycin therapy

**Characteristic**^**a**^	**Total (n = 67)**	**Daptomycin dose (mg/kg)**		
		≤** 8 (n = 41)**	>** 8 (n = 26)**	***P *value**
Pathogen				
MSSA	6 (9.0%)	4 (9.8%)	2 (7.7%)	0.77
MRSA	38 (56.7%)	23 (56.1%)	15 (57.7%)	0.90
Vancomycin MIC ≥ 2 μg/mL	24 (35.8%)	14 (34.1%)	10 (38.5%)	0.80
MRCoNS	10 (14.9%)	6 (14.6%)	4 (15.4%)	0.93
VRE	18 (26.9%)	12 (29.3%)	6 (23.1%)	0.58
Type of infection				
Community-onset	18 (26.9%)	10 (24.4%)	8 (30.8%)	0.57
All bacteremia	54 (80.6%)	32 (78.0%)	22 (84.6%)	0.75
Primary bacteremia	10 (14.9%)	7 (17.1%)	3 (11.5%)	0.54
Endocarditis	11 (16.4%)	3 (7.3%)	8 (30.8%)	0.03
Catheter-related bacteremia	16 (23.9%)	11 (26.8%)	5 (19.2%)	0.48
cSSTI	16 (23.9%)	9 (22%)	7 (26.9%)	0.64
Bone & joint infection	9 (13.4%)	6 (14.6%)	3 (11.5%)	0.72
Other^b^	5 (7.46%)	5 (12.2%)	0 (0%)	0.15
Duration of therapy, days	14 (3–53)	13 (3–53)	14.5 (3–42)	0.47
Mean doses (mg/kg)	7.90	6.98	9.36	

^a^ Data are median (range) for continuous variables.

^b^ Including urinary tract infections (n = 3) and intra-abdominal infections (n = 2).

*cSSTI*, complicated skin and soft-tissue infection; *MIC*, minimum inhibitory concentration; *MRCoNS*, methicillin-resistant coagulase-negative staphylococci; *MRSA*, methicillin-resistant *S. aureus*; *MSSA*, methicillin-susceptible *S. aureus*; *VRE*, vancomycin-resistant enterococci.

Our cohort was heavily-treated before, and daptomycin was used as salvage therapy in 63 patients (94%). Among these patients, the reasons of replacement to daptomycin included clinical or microbiological failure on prior antimicrobial therapy (n = 36), isolates with documented high MIC of glycopeptides (n = 18), and intolerance with grade 3 to 4 toxicities to prior antibiotic therapy (n = 9). Overall, prior use of glycopeptides was found in 59 patients (88.1%). None received gentamicin or rifampin concurrently during daptomycin therapy. There were no differences in baseline underlying diseases, types of infections, disease severity, and causative pathogens between these two groups, except that patients with endocarditis were significantly more likely to receive daptomycin > 8 mg/kg (*P* = 0.03) (Table 2).

The all-cause mortality rate at 14 and 28 day was 16.4% and 35.8%, respectively. The overall in-hospital mortality was 49.3% (33 out of 67 patients) and infection- related mortality (proportionate mortality) was 27.3% (9 out of 33 patients). The other causes of death included healthcare-associated infections due to gram-negative bacteria (n = 10, among which eight were pneumonia and two bacteremia cases) and invasive candidiasis (n = 3), advanced malignancies (n = 6), cardiac events (n = 3), hepatic failure (n = 1), and stroke (n = 1). ICU patients had significantly higher 28-day and in-hospital mortality rates compared to non-ICU patients (46.3% versus 19.2% and 68.3% versus 19.2%, respectively; *p* < 0.05 for both). Overall, 55 (82.1%) patients achieved microbiological success and 52 (77.6%) patients had clinical success. The ICU patients had similar rates of microbiological success (87.8%) and clinical success (80.5%). These outcomes were comparable in the two dosage groups (≤ 8 mg/kg group versus > 8 mg/kg group), even in ICU patients or excluding patients with endocarditis (Table 3). Patients treated with daptomycin due to prior treatment failure had lower rate of clinical success, compared to those treated with daptomycin due to prior adverse drug events (63.9% versus 96.3%, *P* < 0.01). Only one patient with endocarditis had recurrent infection within 30 days after discharge. In multivariate analysis, the significant predictors of in-hospital mortality in 54 patients with bacteremia received daptomycin were under-lying malignancies (adjusted odds ratio [AOR], 21.67; 95% confidence interval [95% CI], 1.94-242.32, *P* = 0.01) and ever ICU stay (AOR, 45.36; 95% CI, 2.07-995.25, *P* = 0.02).

**Table 3:** Outcomes and adverse events of patients with high-dose daptomycin therapy

**Characteristic**	**Total (n = 67)**	**Daptomycin dose (mg/kg)**		
		≤** 8 (n = 41)**	>** 8 (n = 26)**	**P value**
14-day mortality	11 (16.4%)	6 (14.6%)	5 (19.2%)	0.74
28-day mortality	24 (35.8%)	13 (31.7%)	11 (42.3%)	0.44
In-hospital mortality	33 (49.3%)	20 (48.8%)	13 (50.0%)	0.92
ICU patients (n = 41)	28 (68.3%)	17/24 (70.8%)	11/17 (64.7%)	0.74
Infection-related death	9 (13.4%)	5 (12.2%)	4 (15.4%)	0.73
ICU patients (n = 41)	7 (17.1%)	4/24 (16.7%)	3/17 (17.6%)	>0.99
Clinical success	52 (77.6%)	32 (78.0%)	20 (76.9%)	0.91
All bacteremia (n = 54)	43 (79.6%)	27/32 (84.4%)	16/22 (72.7%)	0.32
MRSA (n = 38)	28 (73.7%)	18/23 (78.3%)	10/15 (66.7%)	0.47
Vancomycin MIC ≥ 2 μg/mL (n = 24)	19 (79.2%)	13/14 (92.9%)	6/10 (60%)	0.12
VRE (n = 18)	12 (66.7%)	7/12 (58.3%)	5/6 (83.3%)	0.60
Endocarditis (n = 11)	6 (54.5%)	2/3 (66.7%)	4/8 (50.0%)	>0.99
Catheter-related bacteremia (n = 16)	15 (93.8%)	10/11 (90.9%)	5/5 (100%)	0.59
cSSTI (n = 16)	14 (87.5%)	7/9 (77.8%)	7/7 (100%)	0.48
Bone & joint infection (n = 9)	6 (66.7%)	4/6 (66.7%)	2/3 (66.7%)	>0.99
Microbiological success	55 (82.1%)	36 (87.8%)	19 (73.1%)	0.19
Adverse events				
Cytopenia	47 (70.2%)	28 (68.3%)	19 (73.1%)	0.68
Renal dysfunction	14 (20.9%)	8 (19.5%)	6 (23.1%)	0.73
Elevated AST/ALT	20/66 (30.3%)	12/41 (29.3%)	8/25 (32.0%)	0.82
CPK elevations				
Any	16 (29.2%)	7/37 (18.9%)	9/24 (37.5%)	0.11
By definition	4/61 (6.6%)	0/37 (0%)	4/24 (16.7%)	0.02

*ALT*, alanine aminotransferase; *AST*, aspartate aminotransferase; *CPK*, creatine phosphokinase; *cSSTI*, complicated skin and soft-tissue infection; MIC, minimum inhibitory concentration; *MRSA*, methicillin-resistant *S. aureus*; *VRE*, vancomycin-resistant enterococci.

During daptomycin therapy, the most common adverse events observed were bone marrow suppression (70.2%), abnormal liver aminotransferases levels (29.4%), and renal dysfunction (20.3%). However, these adverse events were generally well tolerated and most of these events could be possibly pronounced by underlying diseases, other medications, or concurrent organ dysfunction, without a direct causal relationship to daptomycin. Overall, three patients discontinued daptomycin due to these adverse events. CPK levels higher than ULN were found in 16 of 61 patients (26.2%). After exclusion of other causes, such as acute coronary syndrome or renal failure, four patients (6.6%) had elevations of CPK level related to use of daptomycin, with the peak CPK levels ranging from 952 to 6528 IU/L. All the four patients with elevations of CPK level had received higher than 8 mg/kg of daptomycin (range, 8.62- 11.11 mg/kg). Three of these events occurred at ≥ 13 days of daptomycin therapy (range, 4–21 days). Only one patient had symptoms of skeletal myopathy. Two of our 67 patients received statins concurrently, but neither had elevations of CPK level by definition. These events resolved after discontinuation of daptomycin. No patients developed eosinophilic pneumonia, which is a rare but well-documented adverse reaction to daptomycin [27].

## Discussion

This cohort study demonstrated that salvage therapy using daptomycin at high doses (> 6 mg/kg to 12 mg/kg) for prior antibiotic treatment failure and intolerance was generally effective and well-tolerated in Taiwanese patients. For use of high-dose daptomycin, only two prospective clinical studies had been published to date [11,15], and all studies were done in western countries (Italy and the United States). To our knowledge, this is the first clinical data describing the use of daptomycin at more than 6 mg/kg in Asian populations. Nearly 90% of our patients received daptomycin following vancomycin or teicoplanin failure, probably owing to the requirement of prospective audit in NTUH. Pre-exposure of glycopeptide might result in elevated MIC of both vancomycin and daptomycin simultaneously [5,24,28-30]. Our results support using higher dose of daptomycin for difficult-to-treat infections caused by resistant pathogens, compatible with other reports [13-17].

The clinical success rates of treatment with high dose daptomycin were 75-94% in the literature [13,15-17]. Bassetti et al. compared patients receiving daptomycin at doses > 6 mg/kg/day and those receiving ≤ 6 mg/kg/day (standard doses) in documented *S. aureus* infections [13]. Both microbiological success (93% versus 68%, *p* < 0.05) and clinical success (94% versus 73%, *P* = 0.05) were significantly superior in the high dose group than the standard dose group. However, the duration of therapy was also significantly longer in the high dose group, which may be a potential limitation. In our cohort, the clinical success rate was 77.6% overall, 80.5% in ICU patients, 79.6% in patients with bacteremia, or 79.2% in patients with MRSA isolates with vancomycin MIC ≥ 2 μg/mL. Compared to patients receiving ≤ 8 mg/kg of daptomycin, those treated with daptomycin > 8 mg/kg did not have better clinical outcomes but had higher rates of CPK level elevations.

The optimal dose of daptomycin is still unknown. In this study, we failed to demonstrate better survival rate, clinical success or microbiological success in patients with daptomycin > 8 mg/kg, compared with those with ≤ 8 mg/kg. It is possible that the physicians in charge tended to prescribe higher doses for sicker patients with more severe infections. However, the baseline characteristics (including Charlson index, Pitt score, ICU stay, and SOFA score) were similar between these two dosing groups, except that more patients with endocarditis were treated with doses > 8 mg/kg. In addition, our small sample size may lack sufficient power to detect small differences between treatment groups. To determine the efficacy of treatment with daptomycin > 8 mg/kg, further investigations in larger cohorts with randomization are warranted.

Approximately 60% of our patients received high-dose daptomycin during ICU admission. This proportion of ICU stay (61.2%) in our cohort is higher than prior reports (25-36%) [17,31]. These ICU patients did not have worse clinical responses or microbiologic outcome despite of higher mortality rate. The efficacy of high-dose daptomycin might be similar in critically-ill patients, and further investigations are needed to confirm our finding.

Overall, defined CPK level elevations occurred in 6.6% of patients in this study. This incidence was similar to the findings from previous studies of high dose daptomycin therapy (3.2-8.3%) [2,13-15,17]. In our study, the incidence of defined CPK level elevations was significantly higher in patients treated with daptomycin > 8 mg/kg (16.7% versus 0%, *p* = 0.02). This finding is not unexpected, since Bhavnani et al. demonstrated that both AUC and minimum serum concentration (C_min_) of daptomycin were significantly associated with CPK elevations. A C_min_ ≥ 24.3 mg/L could lead to a > 30-fold higher risk of CPK elevations. By using Monte Carlo simulations, they also established probabilities of elevated CPK levels stratified by dose [26]. Among our patients with daptomycin > 8 mg/kg, the rate of elevated CPK levels (16.7%) was slightly higher than their prediction (10.7-15.3%). These findings indicate the association of musculoskeletal toxicity with higher doses of daptomycin. Our results also confirmed that CPK level elevations became apparent about 2-week use of daptomycin [26]. Potential adverse events of musculoskeletal toxicity should be alerted if prolonged use of high dose daptomycin, especially after 2 weeks of daptomycin therapy.

Figueroa et al. reported 61 patients treated with a mean dose of 8 mg/kg of daptomycin for a median of 25 days, and 3 patients had musculoskeletal symptoms with grade 3 CPK level elevations (>1000 U/L). All three patients received 8 mg/kg of daptomycin and two of them had body mass index (BMI) class III obesity [14]. In the study from Bhavnani et al., 4 of 6 patients with elevated CPK levels were obese [26]. According to the daptomycin package insert, total body weight (TBW) is the appropriate dosing weight. But dosing daptomycin based on TBW can result in significantly higher maximum serum concentration and AUC in morbidly obese subjects [32]. This could explain why obesity was a risk factor for CPK elevation [26]. However, among our 4 patients with elevations of CPK level, none were obese (BMI range, 17.9-28.7 kg/m^2^; median, 23.4 kg/m^2^). Clinicians should be aware of musculoskeletal toxicity of high-dose daptomycin, even in non-obese patients.

As an observational, uncontrolled, nonrandomized study with a small sample size, there are limitations in our study. First, our study was based on clinical observation, therefore routine follow-up of cultures and CPK levels at regular intervals were not mandatory. Second, we did not check MIC of daptomycin, and only 77.4% of MRSA isolates were checked for MIC of vancomycin. Third, we did not perform pharmacokinetic analyses.

## Conclusion

Our study provided relevant clinical information on high dose (> 6 mg/kg)daptomycin as salvage therapy for Asian populations. High dose daptomycin was effective and well tolerated. Patients treated with doses > 8 mg/kg had a significant higher incidence of elevated CPK levels.

## Competing interests

The authors declare that they have no competing interests.

## Authors’ contributions

CCL and WHS designed, conducted the study and wrote the manuscript. JTW, YCC, YCC, and SCC recruited patients and provided patients care. AC revised the manuscript critically. All authors read and approved the final manuscript.

## Pre-publication history

The pre-publication history for this paper can be accessed here:

http://www.biomedcentral.com/1471-2334/13/66/prepub
